# Supplementary material for: A Facile Method for the Generation of Fe3C Nanoparticle and Fe-Nx Active Site in Carbon Matrix to Achieve Good Oxygen Reduction Reaction Electrochemical Performances
Source: Materials (Basel). 2020 Oct 26;13(21):4779. doi: 10.3390/ma13214779 (PMC7663306; doi:10.3390/ma13214779)
Supplement: Supplementary file 1 [file materials-13-04779-s001.pdf]

Supplementary information

A Facile Method for the Generation of Fe_3C Nanoparticle and Fe-N_x Active Site in Carbon Matrix to Achieve Good Oxygen Reduction Reaction Electrochemical Performances

Calculation of electron transfer number (*n*) for oxygen reduction reaction

For the (rotating ring-disk electrode) RRDE measurements, the disk electrode was scanned at a rate of 10 mV·s^{-1}, and the ring potential was constant at 1.5 V vs RHE. n and HO_2^- yield are calculated by Equations (1) and (2) [1]:

$$n = 4\frac{I_d}{I_d + I_r/N} \tag{1}$$

$$HO_2^- = 200\frac{I_r/N}{I_d + I_r/N} \tag{2}$$

Where I_d is the disk current, I_r is the ring current, and *N* is current collection efficiency of the Pt ring. *N* was determined to be 0.4.

Table S1. Contents (at.%) of N element with different chemical environments calculated from the N 1s XPS spectrum.

Samples	N (at.%) [a]	
	$-NH_2$	Fe$-NH_2$
TA	10.09	-
TA–0.6Fe	6.61	2.11
TA–0.8Fe	5.86	2.55
TA–1.0Fe	6.34	2.19

[a] The different contents of N element (at.%) calculated by the peak areas of -NH_2 and Fe-NH_2.

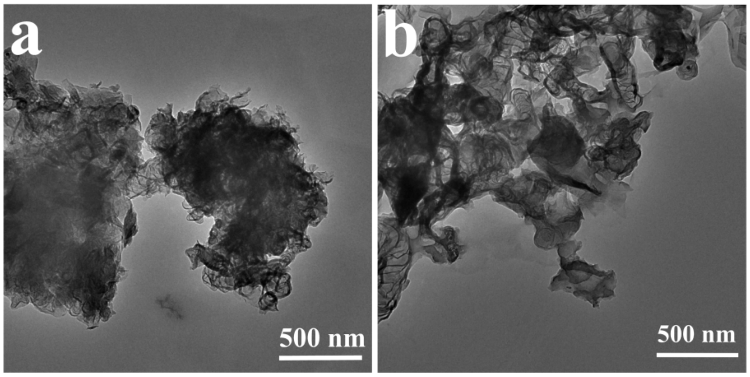

Figure S1. TEM images of CNS–0.6Fe (**a**) and CNS–1.0Fe (**b**).

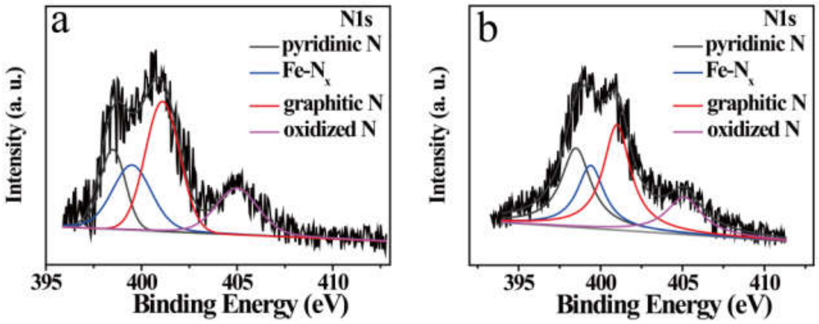

Figure S2. High-resolution XPS spectra of N 1s for CNS–0.6Fe (**a**) and CNS–1.0Fe (**b**).

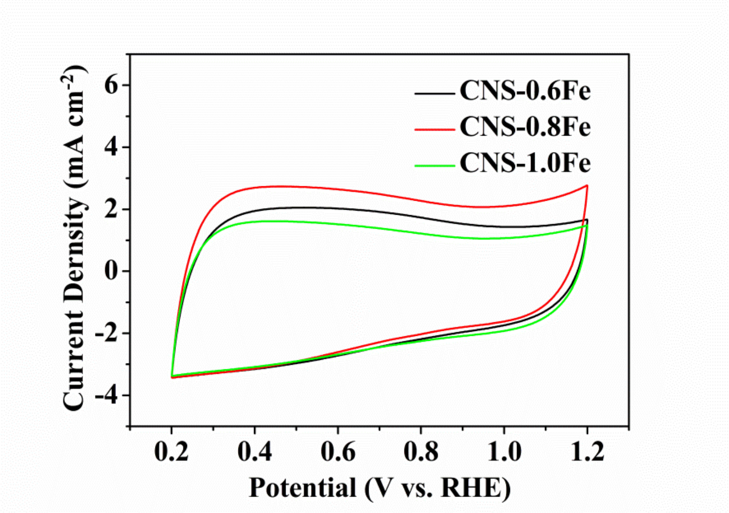

Figure S3. CV curves of CNS–0.6Fe, CNS–0.8Fe and CNS–1.0Fe in Ar-saturated 0.1 M KOH solution, scan rate: 50 V/s.

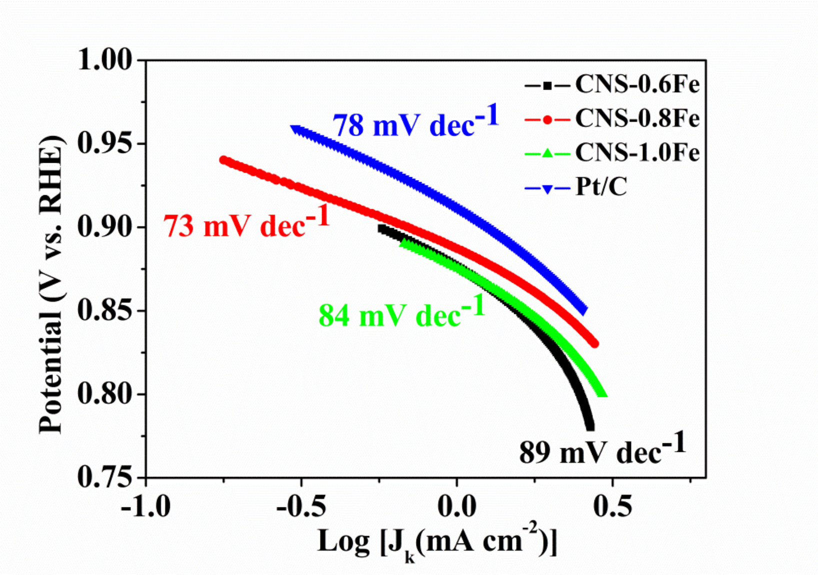

Figure S4. Tafel slopes derived from the LSV curves of CNS–0.6Fe, CNS–0.8Fe, CNS–1.0Fe and Pt/C.

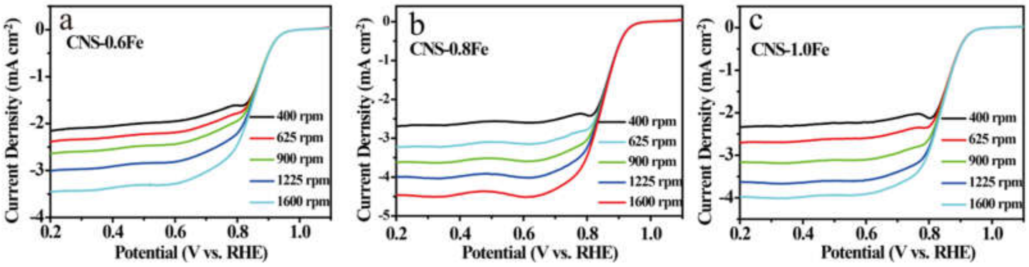

Figure S5. LSV curves of (**a**) CNS–0.6Fe, (**b**) CNS–0.8Fe and (**c**) CNS–1.0Fe at 400–1600 rpm with a scan rate of 10 mV/s in O_2-saturated 0.1 M KOH solution.

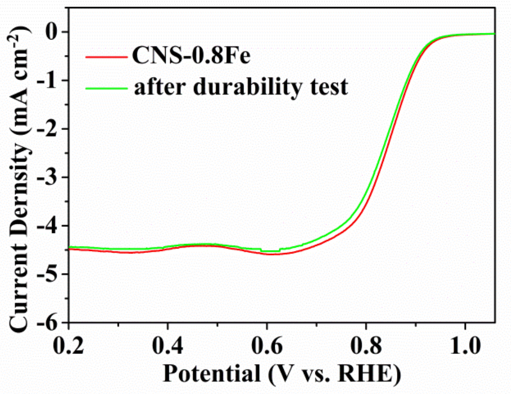

Figure S6. LSV curves of CNS–0.8Fe at 1600 rpm before and after durability test in O_2-saturated 0.1 M KOH solution, scan rate: 50 mV/s.

References

1. Zhang, E.H.; Xie, Y.; Ci, S.Q.; Jia, J.C.; Cai, P.W.; Yi, L.C.; Wen, Z.H. Multifunctional high-activity and robust electrocatalyst derived from metal-organic frameworks. *J. Mater. Chem. A*. **2016**, *4*, 17288–17298.
